# ﻿Two new Cypridopsinae Kaufmann, 1900 (Crustacea, Ostracoda) from southern Africa

**DOI:** 10.3897/zookeys.1076.76123

**Published:** 2021-12-09

**Authors:** Agata Szwarc, Koen Martens, Tadeusz Namiotko

**Affiliations:** 1 Laboratory of Biosystematics and Ecology of Aquatic Invertebrates, Department of Evolutionary Genetics and Biosystematics, Faculty of Biology, University of Gdansk, Wita Stwosza 59, 80–308 Gdansk, Poland University of Gdansk Gdansk Poland; 2 Royal Belgian Institute of Natural Sciences (RBINS), Natural Environments, Vautierstraat 29, 1000 Brussels, Belgium Royal Belgian Institute of Natural Sciences Brussels Belgium; 3 Ghent University, Department of Biology, K.L. Ledeganckstraat 35, 9000 Ghent, Belgium Ghent University Ghent Belgium

**Keywords:** Afrotropical, Cyprididae, microcrustaceans, morphology, taxonomy, temporary waters

## Abstract

Two new Cypridopsinae ostracods, *Potamocyprismeissneri***sp. nov.** and *Sarscypridopsisharundineti***sp. nov.** are described. Both were found only as asexual (all-female) populations in temporary waters of southern Africa. *Potamocyprismeissneri* was collected from a small pan in the North-West Province of South Africa. It is approximately 0.5 mm long and belongs to the species group with long swimming setae on the second antennae. However, the species has a somewhat isolated position in the genus owing to the conspicuously reticulated carapace, which is furthermore densely covered by prominent conuli with normal pores carrying long sensilla, as well as to the wide anterior and posterior flanges on the left valve. To allow identification of the new species in relation to its closest congeners, a key to the species of the genus *Potamocypris* Brady, 1870 from southern Africa is provided. The genus *Sarscypridopsis* McKenzie, 1977 mostly has an Afrotropical distribution with only few species occurring in other regions. *Sarscypridopsisharundineti* was collected from floodplains of the outskirts of the Okavango Delta in Botswana. It is approximately 0.4 mm long and can be distinguished from congeners mainly by the smaller and more oval-shaped valves. We conclude that southern African Cypridopsinae urgently need integrated taxonomic revision, by means of both morphological characters and DNA-sequence data.

## ﻿Introduction

Ostracods, small bivalved crustaceans, have an impressive taxonomic diversity and functional specialisation of their appendages, which are used for locomotion, feeding, and reproduction (Meisch 2000; Smith et al. 2015). They commonly occur in both marine and non-marine habitats, from the oceans and estuaries, over deep lakes to small temporary pools, phytotelmata or troughs, as well as subterranean waters and even semi-terrestrial environments (Mesquita-Joanes et al. 2012; Smith et al. 2015). Ostracoda differ from (most) other crustaceans by a combination of two main features: firstly, by their body oligomerisation with no true body segmentation and secondly, by the strong development of the carapace consisting of two calcified valves hinged along the dorsal margin, and with central adductor muscles attached to the inner part of the valves, crossing the body from one valve to the other and creating characteristic internal muscle scar patterns on them.

Ostracoda are the extant arthropod group with the most abundant fossil record. Although much less diversified than marine lineages, freshwater ecosystems are home to ~ 2300 Recent (living) species and 270 genera (Meisch et al. 2019). Sixteen families have representatives in non-marine habitats, the most diverse family being the Cyprididae Baird, 1845. It includes 24 subfamilies of which the subfamily Cypridopsinae Kaufmann, 1900 is the richest (Meisch et al. 2019) with 22 genera (Savatenalinton 2018; Meisch et al. 2019; Savatenalinton 2020; Almeida et al. 2021). Cypridopsinae are mostly small animals (< 1.0 mm), characterised by a strong reduction of the caudal ramus, which is usually flagellum-like (or even missing) in females, and integrated in the hemipenes in males (Martens and Meisch 1985).

*Potamocypris* Brady, 1870, is after *Cypridopsis* Brady, 1867, the second most abundant genus within the subfamily (Meisch et al. 2019). The 46 species it includes are characterised by 1) asymmetrical valves, with the right valve overlapping the left one dorsally and ventrally, 2) a distinguishing spatula-like shape of the distal palp segment of the maxillula, and 3) a distally tapering caudal ramus. The genus has nearly cosmopolitan distribution, but only 10 species have so far been recorded from the Afrotropical region, and only five from southern Africa (Martens 2001; Meisch et al. 2019).

*Sarscypridopsis* McKenzie, 1977 is mostly distributed in South Africa, with 13 out of the 17 known species described from this country (Sars 1924a, 1924b). Similar to *Potamocypris*, in *Sarscypridopsis* the right valve overlaps the left valve anteriorly, ventrally and posteriorly, but the terminal segment of the maxillular palp is cylindrical and the base of the caudal ramus is triangular (McKenzie 1977).

Here, we describe one species each belonging to *Potamocypris* and to *Sarscypridopsis*. The present paper also constitutes a contribution to the knowledge of the poorly known freshwater ostracod fauna of southern Africa and presents the first comprehensive description of a species of the genus *Sarscypridopsis* with full illustration of valves and appendages.

## ﻿Materials and methods

**Figure 1.**
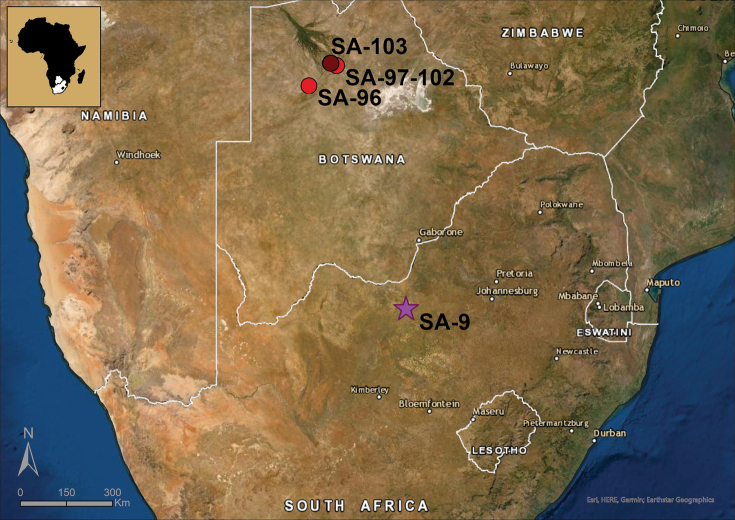
Localities of *Potamocyprismeissneri* sp. nov. (purple star SA-9) in the North-West Province of South Africa and *Sarscypridopsisharundineti* sp. nov. (red dots SA-96 to SA-103) in the outskirts south of the Okavango Delta in Botswana. The type locality of *Sarscypridopsisharundineti* sp. nov. (SA-103) is marked with a dark red dot.

Samples were collected from temporary waters in South Africa and Botswana (Fig. 1) using a hand-net (mesh size of 120 µm) to sweep the bottom surface and among vegetation at the depth of < 50 cm. Sediment samples were rinsed in the field, placed in plastic jars and preserved in 96% ethanol. Physical and chemical properties of the pond water (temperature, pH and electrical conductivity) were measured in situ using a hand-held multi-parameter probe WTW Multi 350i. In the laboratory, samples were thoroughly rewashed with tap water through a 120 µm-mesh sieve, placed in plastic jars and preserved in fresh 96% ethanol. Specimens were sorted, counted, dissected, and mounted using a binocular and light transmission microscope according to Namiotko et al. (2011). Soft parts of dissected ostracods were mounted in glycerin or Hydro-Matrix mounting medium, whereas valves were stored dry on micropalaeontological slides. Species identification was performed using the keys published in McKenzie (1977), Meisch (2000), and Martens (2001), and taxonomical descriptions in Meisch et al. (2019). Drawings of soft parts were made with a camera lucida on a transmission light microscope Nikon Eclipse 50i (Univ. Gdansk, Poland). Carapaces and valves were gold-coated and observed under the scanning electron microscope, Fei Qanta 200 ESEM, at the Royal Belgian Institute of Natural Sciences, Brussels, Belgium as well as under a transmission light microscope Nikon Eclipse 50i (Univ. Gdansk, Poland).

The type specimens are deposited in the Collection of the Royal Belgian Institute of Natural Sciences, Brussels (**RBINS**, general I.D. 3439) and in the Ostracod Collection of the Laboratory of Biosystematics and Ecology of Aquatic Invertebrates, Department of Evolutionary Genetics and Biosystematics, University of Gdansk (**OC-UG**).

Chaetotaxy of the limbs follows the model proposed by Broodbakker and Danielopol (1982), revised for the second antenna by Martens (1987). Names for the limbs were used according to Meisch (2000) except for caudal ramus, which follows Meisch (2007).

### ﻿Abbreviations used in text and figures

**Limbs**:

**A** anterior

**a, a**’ two setae on Pr of T1

**A1** first antenna (antennule)

**A2** second antenna

**alfa (α)** special seta on the 1^st^ podomere of Md palp

**beta (β)** special seta on the 2^nd^ podomere of Md palp

**CR** caudal ramus

**D** distal

**d1**, **d2**, **dp** setae on Pr of T2 or T3

**E** endopod

**e** setae on EI of T2 and T3

**EI**-**EIV** 1^st^ to 4^th^ podomeres of E

**Ex** exterior

**Exo** exopod

**f** setae on EII of T2 and T3

**g** setae on EIII of T2 and T3

**gamma (γ)** special seta on 3^rd^ podomere of Md palp

**GM** (**Gm**) major (minor) claw on EIV of A2

**G1**–**3** anterior and internal claws (or setae) on EIII of A2

**h1**–**3** setae (or claws) on EIV of T2 and T3

**In** interior

**l** large (relative length of setae or claws)

**m** medium (relative length of setae or claws)

**Mastic** masticatory process on Pr of T1

**Md** mandibula

**Mx1** maxillula

**P** posterior

**pl** plumed

**Pr** protopod

**s** small (relative length of setae or claws)

**S1**–**2** plumed setae on 1^st^ podomere of Md palp

**ser** serrated

**T1** first thoracopod (maxilliped)

**T2** second thoracopod (walking leg)

**T3** third thoracopod (cleaning leg)

**t1**–**4** internal setae on EII of A2

**Y** aesthetasc on EI of A2

**y1**–**3** aesthetascs on EII, EIII and EIV of A2 respectively

**ya** aesthetasc on the terminal podomere of A1

**z1**–**3** external setae (or claws) on EIII of A2

**Valves and carapace**:

**Cp** carapace

**H** valve height

**L** valve length

**LV** left valve

**RV** right valve

## ﻿Taxonomy

### Class Ostracoda Latreille, 1802

**Subclass Podocopa Sars, 1866**

**Order Podocopida Sars, 1866**

**Suborder Cypidocopina Baird, 1845**

**Superfamily Cypridoidea Baird, 1845**

**Family Cyprididae Baird, 1845**

**Subfamily Cypridopsinae Kaufmann, 1900**

### Genus *Potamocypris* Brady, 1870

#### ﻿ Potamocypris meissneri sp. nov.

Taxon classificationAnimaliaPodocopidaCyprididae

76208F96-DF78-557C-BEF7-6296826C63AF

http://zoobank.org/03D4496-F0A1-4C66-B481-BFDE1F939B4D

Figures 2 , 3 , 4

##### Material examined.

**Type locality**: South Africa, North-West Province, small temporary open pan (SA-9) near the village of Ganalaagte (Fig. 1, Suppl. material 1: Fig. 1A); 26°26'45"S, 25°32'19"E, elevation 1380 m a.s.l.; 1 Apr. 2011; T. Namiotko leg.

***Holotype***: • 1 ♀ (adult); dissected female stored on a permanent microscopic slide and valves stored dry on a micropalaeontological slide (RBINS INV.159058). ***Paratypes***: South Africa • 2 ♀♀ (adults); same collection data as for holotype (OC-UG 110401-9A2L and OC-UG 110401-9A3L) • 136 ♀♀ (adults), 78 juv.; same collection data as for holotype: 115 ♀♀ and 78 juv. preserved in 96% ethanol; 16 ♀♀ stored as the holotype; 5 ♀♀ stored with carapaces stored on micropalaeontological slides (RBINS INV.159059–INV.159063); repositories: RBINS and OC-UG. **Accompanying ostracod fauna**: Hemicypriscf.inversa (Daday, 1913); Limnocytherecf.stationis Vávra, 1891.

##### Etymology.

This species is named after Dr Włodzimierz Meissner, Professor of ornithology at the University of Gdansk, Poland, a long-standing friend of TN who provided unrelenting support in the collection of ostracods from all over the world and who has encouraged and helped TN to join various scientific expeditions for collecting ostracods.

##### Diagnosis.

Carapace in lateral view somewhat ovoid, broadly rounded dorsally, with both extremities more or less equally rounded, ventral margin weakly concave, and maximum height situated at mid-length. Valves distinctly asymmetrical, with LV overlapping RV anteriorly and posteriorly, RV overlapping LV dorsally and ventrally. Anterior and posterior margins on LV with marginal flange, anterior one larger than posterior one. Carapace external surface hirsute, strongly ornamented with ridges, set with thickly rimmed pores with long sensilla. Antenna with long swimming setae. Terminal segment of maxillula palp spatulate with five claws. T1 with two hirsute branchial rays. CR of whip-like shape with elongated base, fused with distal long flagellum-like seta and set with additional short subapical seta.

##### Description.

**Figure 2.**
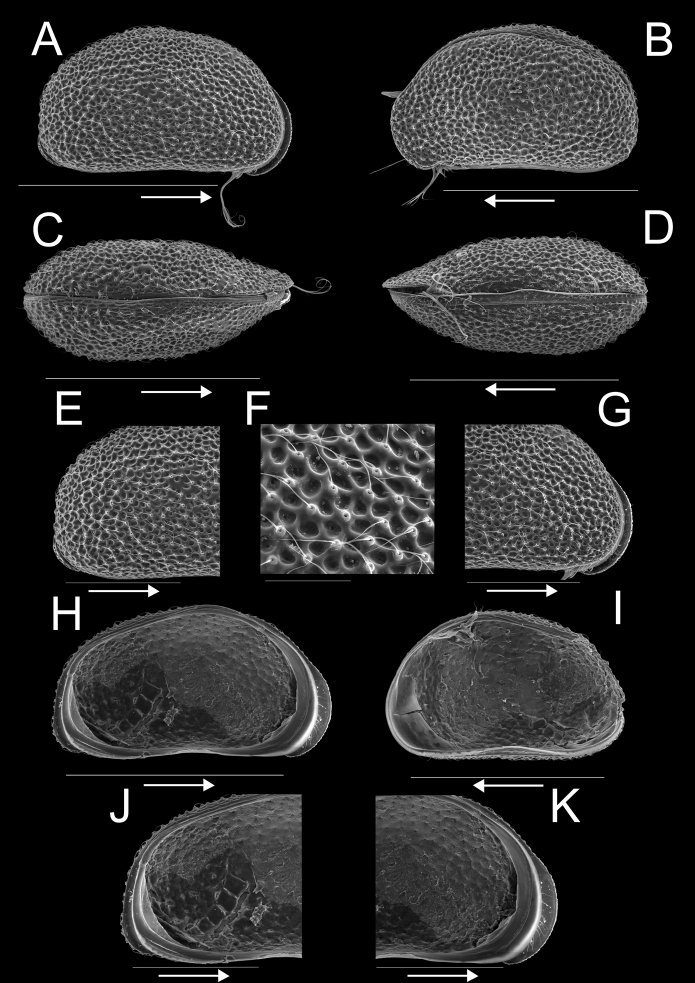
Carapace and valves of *Potamocyprismeissneri* sp. nov. ♀ **A**RBINS INV.159060 **B**RBINS INV.159062 **C**RBINS INV.159061 **D**RBINS INV.159063 **E–G**RBINS INV.159060 **H–K**RBINS INV.159059. **A** carapace right view **B** carapace left view **C** carapace dorsal view **D** carapace ventral view **E** carapace right view of posterior end, detail of A **F** detail of external surface of A **G** carapace right view of anterior end, detail of A **H** left valve internal view **I** right valve internal view **J** left valve internal view of posterior part, detail of H **K** left valve internal view of anterior part, detail of H. Scale bars: 400 μm (**A–D, H, I**); 200 μm (**E, G, J, K**); 50 μm (**F**); arrows indicate anterior end.

**Female.**Cp in lateral view (Fig. 2A, B) with posterior extremity more broadly rounded than anterior one, dorsal margin broadly rounded, ventral margin weakly concave, almost straight. Maximum height situated at mid-length. Valves distinctly asymmetrical, with LV overlapping RV along anterior and posterior margins with flanges (Fig. 2E, G). Carapace of hirsute appearance with reticulate external surface bearing numerous thickly rimmed normal pores with long sensilla (Fig. 2F). Cp in dorsal (Fig. 2C) and ventral view (Fig. 2D) with posterior extremity slightly rounded, anterior extremity more pointed. Greatest width situated slightly behind mid-length. RV slightly overlapping LV dorsally and ventrally. LV in internal view (Fig. 2H) subtriangular, with greatest height situated in front of mid-length, posterior part of dorsal margin straight and sloping towards the posterior side; anterior margin rounded, posterior margin almost straight, ventral margin slightly sinuous at mid-length. Anterior and posterior margins with marginal flanges, extending beyond inwardly displayed selvage along anterior and posterior margins, but peripheral along ventral margin (Fig. 2H, J–K). Flanges particularly expanded in the lower two-thirds of the anterior and posterior margins, with LV overlapping RV (Fig. 2A). Anterior and posterior calcified inner lamella narrow with one inner list, the latter not reaching halfway posterior margin. RV in internal view (Fig. 2I) ovoid, with maximum height situated in front of mid-length, anterior margin rounded, posterior margin less so. Anterior calcified inner lamella wide without inner list, but with submarginal peripheral selvage.

**Figure 3.**
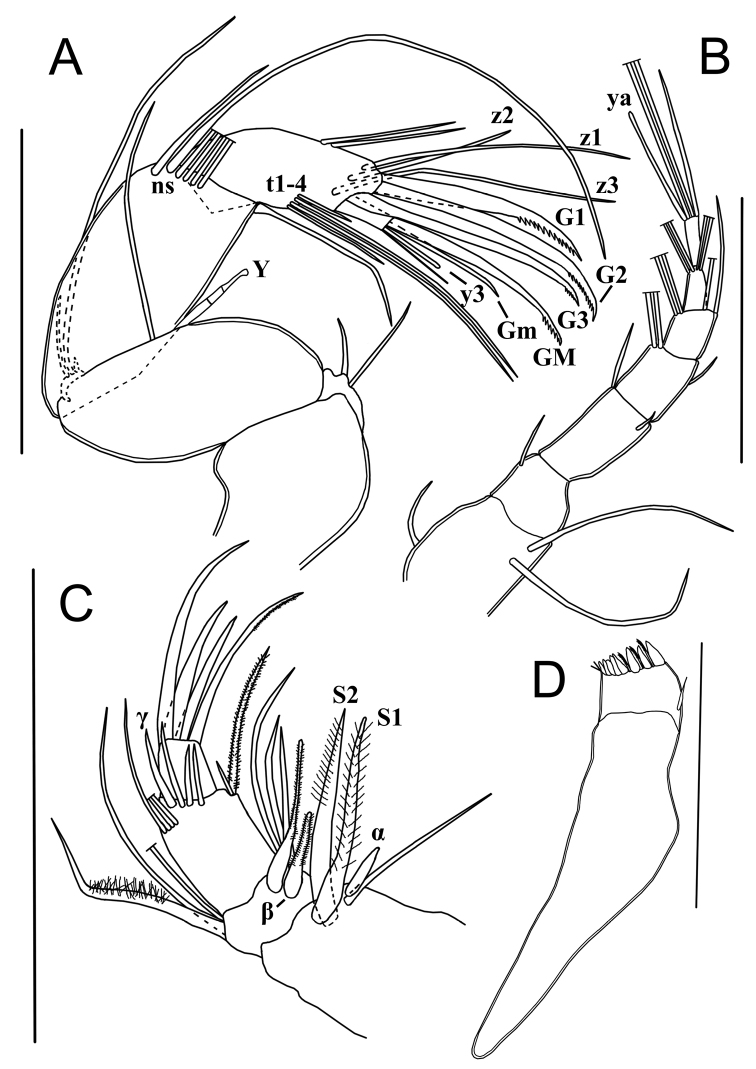
Limbs of *Potamocyprismeissneri* sp. nov. ♀. Holotype (OC-UG 110401-9A1L) **A** second antenna **B** first antenna **C** mandibular palp **D** mandibular coxa. Scale bars: 100 μm. Abbreviation: ns = natatory setae.

A1 (Fig. 3B) 7-segmented. First segment with one short subapical dorsal seta (not reaching tip of segment) and two long ventral setae. Second segment subquadrate with one short antero-dorsal seta. Rome organ not seen. Third segment ~ 2× as long as wide, with two setae, one short antero-dorsal (reaching tip of next segment) and one very short antero-ventral seta. Fourth segment with two long antero-dorsal setae and one short antero-ventral seta (reaching 1/3 of next segment). Fifth segment bearing two long antero-dorsal setae and two ventral setae, one long and one of medium length (reaching beyond tip of terminal segment). Penultimate segment with four long apical setae. Terminal segment distally with three (two long and one medium-length) setae and an aesthetasc ya, length of aesthetasc ya ~ 5/6 of that of medium seta.

Chaetotaxic formula: I: A-1s, P-2l / II: A-1s / III: A-1s, P-1s / IV: A-2l, P-1s / V: A-2l, P-1l-1m / VI: A-4l / VII: D: 2l-1m-ya.

A2 (Fig. 3A) with protopodite, exopodite and 3-segmented endopodite. Basal segment of protopodite with two short ventro-apical setae. Second segment of protopodite with one long apical seta, reaching beyond first endopodal segment. Exopodite reduced to a small plate with three setae, two short and one long, the latter reaching halfway second endopodal segment. Endopodite 3-segmented. First endopodal segment with one long ventro-apical seta, extending beyond tip of terminal segment and one aesthetasc Y of medium length, divided in three parts, distal sensorial part with conspicuously sunken appearance; antero-dorsal with five long natatory setae (reaching tips of terminal claws) and one shorter (6^th^) seta reaching half of next segment. Second endopodal segment undivided, with two subequal medio-dorsal setae and four medio-ventral setae (t1-t4), two long, one medium and one short; distally with three z-setae, z1 and z3 long, z2 ~ 1/2 the length of z1 and z3, and three long serrated G-claws: G1 thick and apically strongly serrated, G2 and G3 more slender. Terminal endopodal segment subquadrate, with a long serrated claw GM, a shorter (~ 2/3 length of GM) smooth claw Gm and an aesthetasc y3 fused with slightly longer accompanying seta. Aesthetascs y1, y2 and seta g not seen, the latter almost certainly absent as typical of the subfamily.

Chaetotaxic formula: Pr: 1l / Exo: 1l-2s / EI: A-5l-1m, P: Y-1l / EII+III: A-2m, P-1m(t1)-2l(t2,3)-1s(t4), D-2l(z1,z3)-1m(z2)-3l(G1,2,3: ser) / EIV: 1l(GM: ser)-1m(Gm)-y3–1m

**Figure 4.**
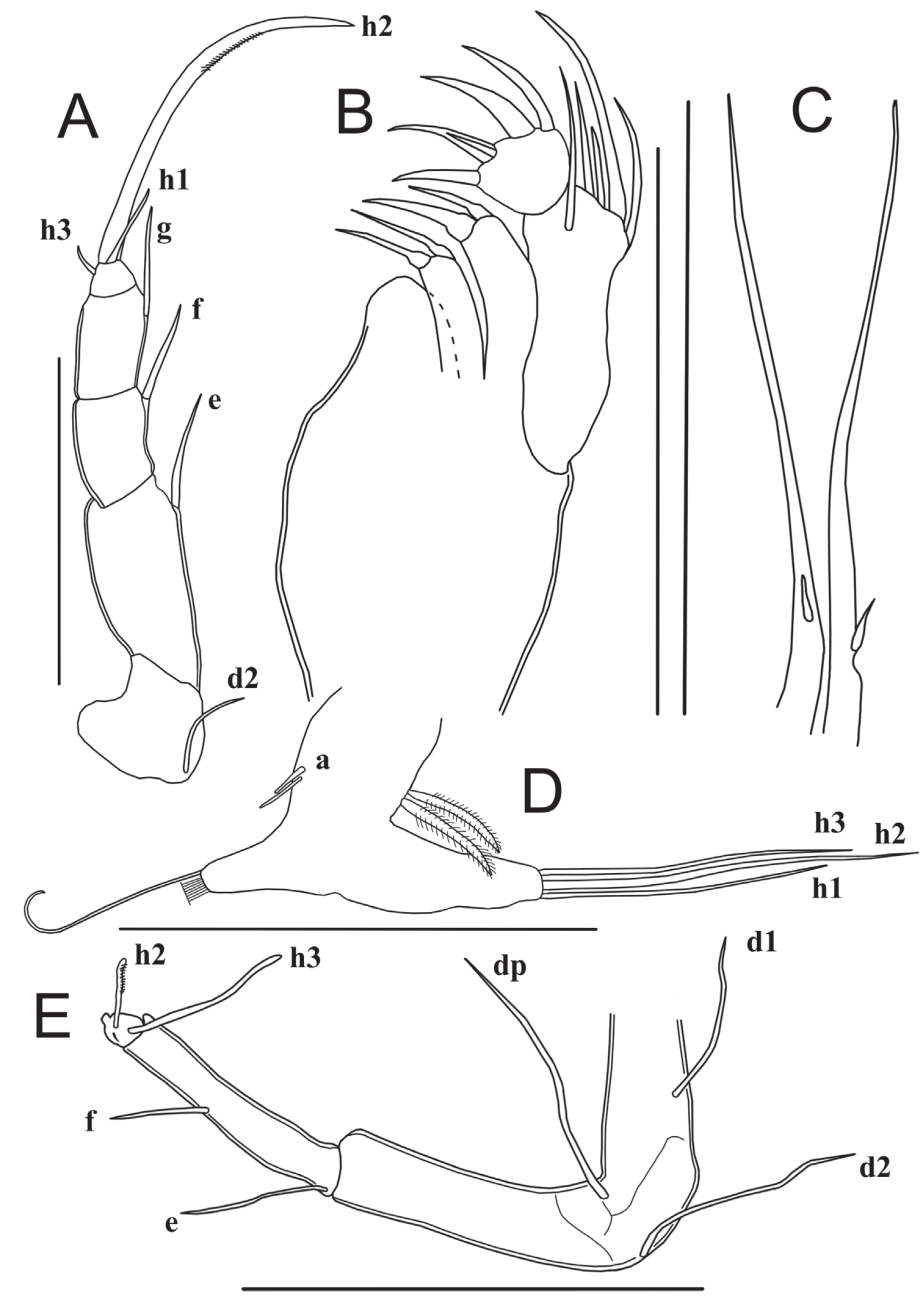
Limbs of *Potamocyprismeissneri* sp. nov. ♀ **A, B, C** holotype (OC-UG 110401-9A1L) **D** paratype (OC-UG 110401-9A3L) **E** paratype (OC-UG 110401-9A2L) **A** second thoracopod (walking leg) **B** maxillula **C** caudal ramus **D** first thoracopod (maxilliped) **E** third thoracopod (cleaning leg). Scale bars: 100 μm.

Md with sclerotised coxa (Fig. 3D) and 4-segmented palp (Fig. 3C). First palp-segment ventro-apically with two long plumed setae (S1 and S2), one long slender and smooth seta and a short but stout, smooth α-seta. Second segment antero-dorsally with two long slender and smooth setae and one thick plumed seta; ventrally with three unequally long smooth setae, one long hirsute seta and a stout and hirsute β-seta. Third segment antero-ventrally with one long hirsute seta and one short smooth seta; medio-dorsally with four setae, two reaching tip of terminal segment, and two longer setae, one of these smooth γ-seta; antero-dorsally with four setae reaching beyond tips of terminal segment. Terminal segment with four claws, two ~ 3× as long as length of terminal segment, one of these serrated, and two shorter claws. Md coxa typically elongated, distally with rows of teeth and small setae, and with one short smooth seta situated near the insertion place of the palp.

Chaetotaxic formula: Palp: I: In-1s(alfa)-1l-2l(S1,S2: pl) / II: In-1s(beta: pl)-1l(pl)-2m-1l, Ex:1l(pl)-2l / III: In-1l(pl)-1s, D-3m-1m(gamma), Ex-4m / IV: 2m-1l-1l(ser)

Mx1 (Fig. 4B) with three endites (with chaetotaxy incompletely illustrated), a 2-segmented palp and a large respiratory plate (not illustrated). Third endite with two smooth teeth bristles (Zahnborsten). First palp-segment dorso-apically with four unequal setae; medio-dorsally with one long, subapical seta, reaching beyond tip of terminal segment. Second palp-segment spatulate, apically with four stout claws (~ 1.5× as long as terminal segment) and one shorter claw (~ 1/2 length of others).

Chaetotaxic formula: Palp: I: Ex-3s-1l, In-1m / II: D-5s

T1 (Fig. 4D). Protopodite with two short a-setae; b-, c-, and d-setae absent, endite distally with a dozen apical setae (exact number not determined). Endopod elongated, apically with one long seta (h2) and two shorter setae (h1 and h3) of differing lengths. Respiratory plate with two hirsute rays.

Chaetotaxic formula: Pr: A-2s(a and a’) / Mastic: D-? / Exo: 2m(pl) / E: D-3l

T2 (Fig. 4A) a walking limb. Protopodite with seta d2 of medium length, seta d1 absent. First three endopodal segments each with one ventro-apical seta. Setae e and f reaching tip of the next segment, seta g ~ 3× as long as terminal segment. Fourth segment with one short seta (h3), one medium seta of length (h1) and a long claw, distally serrated (h2), the latter ~ 3× as long as the second endopodal segment.

Chaetotaxic formula: Pr: A-1s(d2) / EI: A-1m / EII: A-1m / EIII: A-1m / EIV: P-1s(h3), D-1m(h1)-1l(h2 G:ser)

T3 (Fig. 4E) a cleaning limb. First segment with two long apical setae (dp and d2) and one shorter medio-ventral seta (d1). Second segment fused with third segment, with e-seta of medium length, and with f-seta reaching tip of segment. Distal part of limb consisting of a pincer-organ (fusion between tip of third segment and fourth segment), bearing a seta (h3) of medium length and a short, serrated seta (h2); h3 ~ 2/3 of the length of penultimate segment, h2 ~ 1/3 of length of h3.

Chaetotaxic formula: Pr: A-1l(d2)-1m(d1), P-1l(dp) / EI: A-1m(e) / EII + III: A-1s(f) / EIV: 1s(h2: ser)-1m(h3)

CR (Fig. 4C). Reduced to a whip-like structure, with elongated base fused with long flagellum-like seta and bearing a short subapical seta.

##### Remark.

It should be noted that juveniles of this species do not have tubercles on the valves (see discussion).

**Measurements** (in μm). Cp (n = 4): L = 512–526, H = 305–306; LV (n = 11): L = 510–530, H = 278–298; RV (n = 11): L = 498–517, H = 301–319.

**Male** unknown.

##### Ecology.

*Potamocypismeissneri* was collected only from the type locality in the North-West Province of South Africa. This is an open temporary pan with the following physical and chemical water properties: pH = 7.0, electrical conductivity = 36 µS/cm and water temperature 25.8°C.

###### ﻿Key to southern African *Potamocypris* species (partly based on Martens 2001):

**Table:** 

1	Natatory setae of A2 short (not reaching tips of terminal claws	***P.paludum* Gauthier, 1939**
–	Natatory setae of A2 long	**2**
2	Cp elongated (L ≥ 2× H), crescent-shaped	***P.mastigophora* (Methuen, 1910)**
–	Cp compressed (L < 2× H) , differently shaped, not crescent-shaped	**3**
3	Cp subtriangular, dorsally arched with blunt angle	***P.gibbula* (Sars, 1924)**
–	Cp with dorsal margin broadly rounded or straight on a long distance and sloping down to the posterior	**4**
4	Cp with posterior margin rounded; maximum height at mid-length	***P.meissneri* sp. nov.**
–	Cp with posterior margin straight; maximum height in front of mid-length	**5**
5	RV with wide dorsal overlap of LV	***P.deflexa* (Sars, 1924)**
–	Dorsal overlap of RV minute or lacking	***P.humilis* (Sars, 1924)**

### Genus *Sarscypridopsis* McKenzie, 1977

#### ﻿ Sarscypridopsis harundineti sp. nov.

Taxon classificationAnimaliaPodocopidaCyprididae

29445D1B-03AD-5495-8756-025095AA29AD

http://zoobank.org/45D19D9C-FD3E-4D1E-8C2C-65F14004373F

Figures 5 , 6 , 7

##### Material examined.

**Type locality**: Botswana, North-West District, floodplains south of Okavango Delta (SA-103); grassy shore of seasonal pond near the city of Maun (Fig. 1, Suppl. material 1: Fig. 1B), 19°52'12"S, 23°20'23"E, elevation ca. 940 m a.s.l.; 15 Sept. 2012; T. Namiotko leg.

***Holotype***: • 1 ♀ (adult); dissected female stored on a permanent microscopic slide and valves stored dry on a micropalaeontological slide (RBINS INV.159064). ***Paratypes***: BOTSWANA • 27 ♀♀ (adults); same data as for holotype; 2 ♀♀ stored as the holotype (OC-UG 120915-3A2L, 120915-3A3L); 22 ♀♀ preserved in 96% ethanol (120915-30); 3 ♀♀ stored on micropalaeontological slides (RBINS INV.159065–INV.159067); repositories: RBINS and OC-UG. **Accompanying ostracod fauna**: *Heterocyprisoblonga* (Sars, 1924); Limnocytherecf.stationis; *Plesiocypridopsisnewtoni* (Brady and Robertson 1870).

##### Additional material.

Botswana – North-West District: • **SA-96** (Fig. 1, Suppl. material 1: Fig. 1C): 1 juv.; endorheic Lake Ngami; 20°28'57"S, 22°42'08"E; elevation ca. 930 m a.s.l.; 12 Sept. 2012; accompanying ostracod fauna: *Hemicyprisinversa*; *Heterocyprisgiesbrechti* (G.W. Müller, 1898) • **SA-97** (Fig. 1, Suppl. material 1: Fig. 1D): 11 ♀♀ and 1 juv.; Thamalakane river near the city of Maun; 19°55'52"S, 23°30'38"E; elevation ca. 940 m a.s.l.; 13 Sept. 2012; accompanying ostracod fauna: *Candonopsisnavicula* Daday, 1910; *Chrissiaperarmata* (Brady, 1904); *Heterocyprisoblonga*; Isocypriscf.priomena G.W. Müller, 1908; Limnocytherecf.stationis; Physocypriacf.capensis (Sars, 1895); *Potamocyprismastigophora* (Methuen, 1910); Sarscypridopsiscf.elizabethae (Sars, 1924); *Sclerocypris* sp., *Stenocyprismalayica* Victor & Fernando, 1981; Strandesiacf.prava Klie, 1935 • **SA-98** (Fig. 1, Suppl. material 1: Fig. 1E): 6 ♀♀; floodplains south of Okavango Delta, temporary channel near the city of Maun; 19°52'15"S, 23°21'06"E; elevation ca. 940 m a.s.l.; 14 Sept. 2012; accompanying ostracod fauna: *Heterocyprisgiesbrechti* • **SA-99** (Fig. 1, Suppl. material 1: Fig. 1F): 16 ♀♀ and 1 juv.; floodplains south of Okavango Delta, temporary channel near the city of Maun; 19°52'15"S, 23°20'45"E; elevation ca. 940 m a.s.l.; 14 Sept. 2012; accompanying ostracod fauna: *Heterocyprisoblonga*; *Potamocyprisdeflexa* (Sars, 1924); *Potamocyprismastigophora*; *Zonocypriscostata* (Vávra, 1897) • **SA-100** (Fig. 1, Suppl. material 1: Fig. 1G): 11 ♀♀; floodplains south of Okavango Delta, flooded swamp and grassland near the city of Maun; 19°52'04"S, 23°20'38"E; elevation ca. 940 m a.s.l.; 14 Sept. 2012; accompanying ostracod fauna: *Heterocyprisoblonga*; *Stenocyprismalayica*; *Zonocypristuberosa* G.W. Müller, 1908 • **SA-101** (Fig. 1, Suppl. material 1: Fig. 1H): 6 ♀♀; floodplains south of Okavango Delta, isolated pool in flooded grassland near the city of Maun; 19°51'39"S, 23°19'41"E; elevation ca. 940 m a.s.l.; 15 Sept. 2012; accompanying ostracod fauna: *Heterocyprisoblonga* • **SA-102** (Fig. 1, Suppl. material 1: Fig. 1I): 1 ♀; floodplains south of Okavango Delta, floodplain channel near the city of Maun; 19°52'06"S, 23°20'41"E; elevation ca. 940 m a.s.l.; 15 Sept. 2012; accompanying ostracod fauna: *Heterocyprisoblonga*.

All individuals collected by T. Namiotko; 51 ♀♀ and 3 juv. are stored in 96% ethanol and 3 ♀♀ are stored as holotype. Repositories: RBINS and OC-UG.

##### Etymology.

This species is named after the term “reed-bed” (Latin: *harundinetum*), the original meaning of the name of the town Maun in Botswana close to the sites from where *Sarscypridopsisharundineti* was collected. The name Maun is derived from the language of Bantu-speaking people and translates as “the place of river reeds”.

##### Diagnosis.

Carapace in lateral view with anterior and posterior margins nearly symmetrically rounded, dorsal margin almost evenly rounded with greatest height situated just behind mid-length, ventral margin almost straight. RV overlapping LV anteriorly, posteriorly and ventrally, LV slightly overlapping RV dorsally. Carapace surface smooth (with fine reticulation in the central area), with rare thickly rimmed normal pores with short sensilla, situated mostly in the posterior and postero-dorsal parts. Antenna with long swimming setae, and supporting aesthetasc Y with distinctive distal bulbous sensory part. Terminal segment of maxillular palp elongated, ~ 2× as long as wide, bearing four long claws. T1 with four branchial rays. CR reduced, with elongated, triangular base.

##### Description.

**Figure 5.**
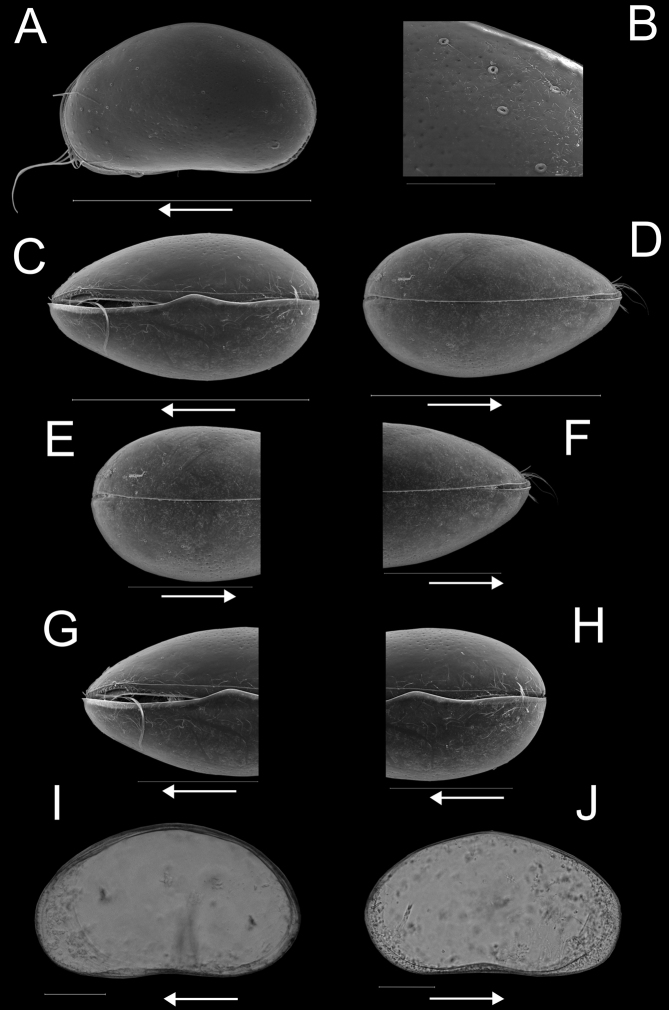
Carapace and valves of *Sarscypridopsisharundineti* sp. nov. ♀ **A, B**RBINS INV.159066 **C**RBINS INV.159067 **D, E, F**RBINS INV.159065 **G, H**RBINS INV.159067 **A** carapace left view **B** detail of external surface of A **C** carapace ventral view **D** carapace dorsal view **E** carapace posterior part, detail of D **F** carapace anterior part, detail of D **G** carapace anterior part, detail of C **H** carapace posterior part, detail of C **I** left valve external view **J** right valve external view Scale bars: 400 μm (**A, C, D**); 50 μm (**B**); 200 μm (**E–H**); 100 μm (**I, J**); arrows indicate anterior end.

**Female.**Cp in left lateral view (Fig. 5A) with anterior and posterior margins nearly symmetrically rounded, dorsal margin almost evenly rounded, with greatest height situated just behind mid-length; ventral margin almost straight. RV overlapping LV anteriorly and posteriorly. LV slightly overlapping RV dorsally, RV overlapping LV ventrally (Fig. 5E–H). External surface smooth with fine reticulation in the central area; with rare, thickly rimmed pores with extending sensilla situated mostly in the anterior and postero-dorsal parts (Fig. 5B). Cp in dorsal (Fig. 5D) and ventral views (Fig. 5C) sub-elliptical, lateral margins unevenly rounded, widening posteriorly; posterior edge broadly rounded, anterior one more pointed. Greatest width situated behind mid-length. LV in internal view (Fig. 5I) ovoid, with greatest height situated at mid-length. Anterior and posterior margins almost equally rounded, ventral margin slightly sinuous at mid-length. Anterior and posterior calcified inner lamella narrow with marginal selvage. RV in internal view (Fig. 5J) with posterior margin broadly rounded, anterior margin more pointed and with ventral margin almost straight. Anterior and posterior calcified inner lamella wider than on LV; no selvage or inner list.

**Figure 6.**
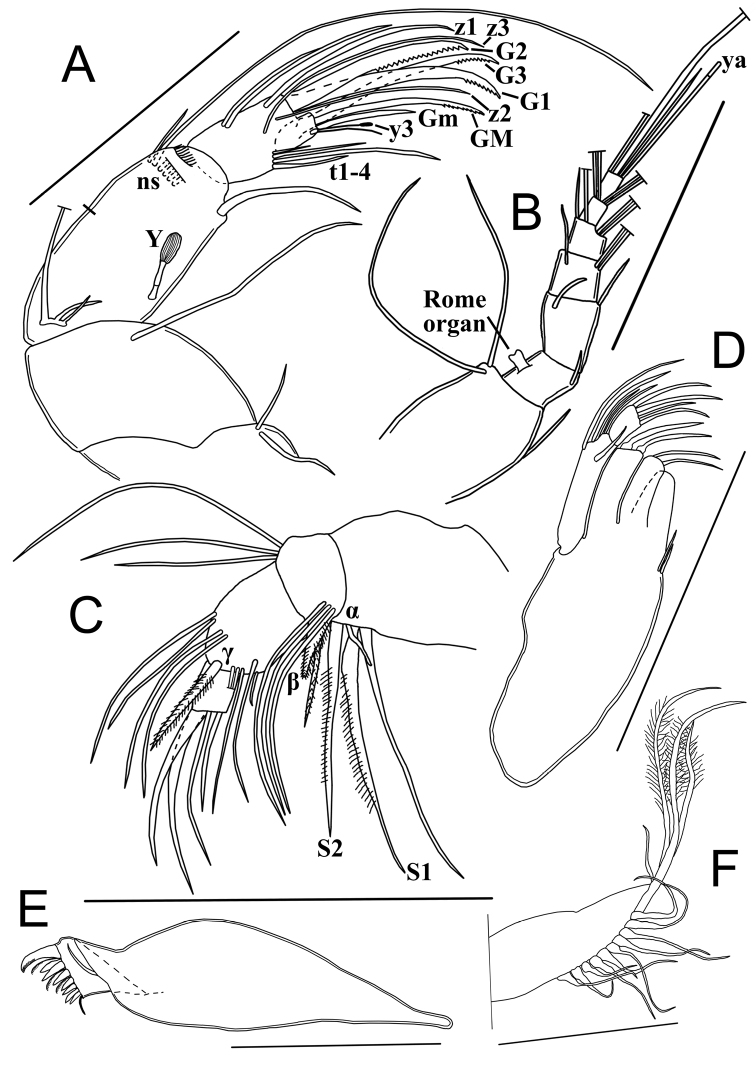
Limbs of *Sarscypridopsisharundineti* sp. nov. ♀. **A, B, E, F** holotype (OC-UG 120915-3A1L) **C, D** paratype (OC-UG 120915-3A3L) **A** Second antenna **B** First antenna **C** Mandibular palp **D** Maxillula **E** Mandibular coxa **F** Maxillular respiratory plate. Scale bars: 100 μm. Abbreviation: ns = natatory setae.

A1 (Fig. 6B) 7-segmented. First segment with one short subapical dorsal seta (reaching beyond tip of segment) and two long ventro-apical setae. Second segment with one short dorso-apical seta (reaching 1/3 of length of next segment) and a large ventral Rome organ. Third segment ~ 0.5× as long as wide, with two apical setae, one short dorsal seta (reaching beyond tip of next segment) and one short ventral seta (not reaching tip of next segment). Fourth segment with two long dorso-apical setae and one short ventro-apical seta (reaching 1/3 of penultimate segment). Fifth segment bearing two long ventro-apical setae and two dorso-apical setae, one long and one short (the latter nearly reaching tip of terminal segment). Penultimate segment with four long apical setae. Terminal segment with three (two long and one medium) setae and one aesthetasc ya, the latter slightly longer than the seta of medium length.

Chaetotaxic formula: I: A-1s, P-2l / II: A-1s, P-r / III: A-1s, P-1s / IV: A-2l, P-1s / V: A-2l, P-1l-1s / VI: A-4l / VII: D: 2l-1m-ya.

A2 (Fig. 6A) with protopodite, exopodite and 3-segmented endopodite. Basal segment of protopodite with two short ventro-apical setae. Second segment of protopodite with one long ventro-apical seta. Exopodite reduced to a small plate with three setae, two short and one long. Endopodite 3-segmented. First endopodal segment with long ventro-apical seta, extending beyond tip of terminal segment; a large aesthetasc Y with a distinct distal bulbous sensory part; dorso-apically with five long natatory setae (reaching far beyond tips of terminal claws) and one shorter (6^th^) seta reaching 1/3 of length of next segment. Second endopodal segment undivided, medio-dorsally with two subequally long setae and medio-ventrally with four unequal setae (t1-t4), one long and three short; distally with three long z-setae (z1, z2, z3) and three long serrated G-claws: G2 thick and apically strongly serrated, G1 and G3 more slender. Terminal (third) endopodal segment subquadrate with a long serrated claw GM, a shorter (~ 1/2 length of GM) smooth claw Gm and an aesthetasc y3 fused with slightly longer accompanying seta. Aesthetascs y1, y2 and seta g not seen, the latter almost certainly absent as typical of the subfamily.

Chaetotaxic formula: Pr: 1l / Exo: 1l-2s / EI: A-5l-1s, P-Y-1l / EII+III: A-2l, P-1s(t1)-1l(t2)-2s(t3,4), D-3l(z1,z2,z3)-3l(G1,2,3: ser) / EIV: 1l(GM: ser)-1m(Gm)-y3–1s

Md with sclerotised coxa (Fig. 6E) and 4-segmented palp (Fig. 6C). Md-coxa elongated, distally with rows of teeth and small setae, and with one short, smooth seta situated near the palp. First palp-segment ventro-apically with two long plumed setae (S1 and S2), one long, slender seta and a short smooth α-seta, situated in between the two S-setae. Second segment dorso-apically with three unequally long slender setae; ventrally with three long, smooth setae, one medium hirsute seta and hirsute, cone-shaped β-seta. Third segment ventro-apically with one long and one short seta; medio-apically with three setae, all reaching tip of terminal segment and a hirsute and long γ-seta; dorso-apically with four setae, all extending far beyond tips of terminal segment. Terminal segment bearing four claws, three ~ 4× as long as terminal segment and one shorter.

Chaetotaxic formula: Palp: I: In-1s(alfa)-1l-2l(S1,S2: pl) / II: In-1s(beta: pl)-1m(pl)-3l, Ex-3l / III: In-1l-1s, D-3l-1l(gamma: pl), Ex-4l / IV: 3l-1m

**Figure 7.**
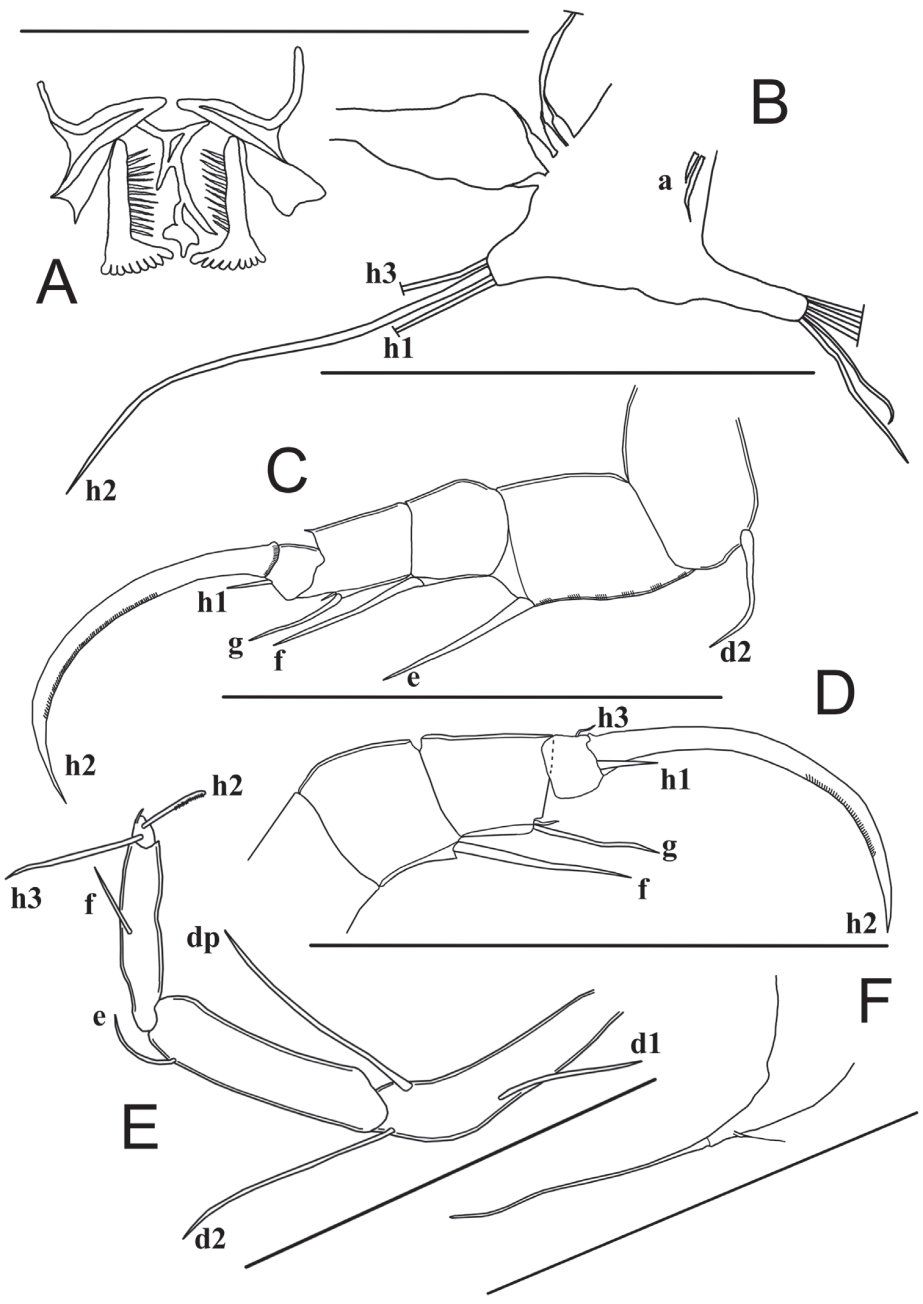
Limbs of *Sarscypridopsisharundineti* sp. nov. ♀ **A, B, C, F** paratype (OC-UG 120915-3A3L) **D** paratype (OC-UG 120915-3A2L) **E** holotype (OC-UG 120915-3A1L) **A** Food-rake **B** First thoracopod (maxilliped) **C** Second thoracopod (walking leg) **D** Second thoracopod distal end **E** Third thoracopod (cleaning leg) **F** Caudal ramus. Scale bars: 100 μm.

Rake-like organs (food-rakes) (Fig. 7A) T-shaped, each with nine apical teeth.

Mx1with three endites and 2-segmented palp (Fig. 6D) and a large respiratory plate (Fig. 6F). First endite with two short setae near its base. Third endite with two smooth teeth bristles. First palp-segment with four unequal dorso-apical setae, one long, one medium and two short; medio-apically with one seta (reaching 1/2 length of terminal segment). Second segment elongated (~ 2× as long as wide), apically with four unequal but long claws (~ 2× as long as terminal segment). Respiratory plate large and elongate, distally with a row of more than eleven smooth rays and three long plumose rays.

Chaetotaxic formula: Palp: I: Ex-2s-1m-1l, In-1s / II: D-4m

T1 (Fig. 7B). Protopodite with two short unequal a-setae; b-, c- and d-setae absent, endite distally with a dozen apical setae (exact number not determined). Endopod elongated, apically with one long seta (h2) and two shorter setae (h1 and h3). Respiratory plate with four long rays with swollen bases.

Chaetotaxic formula: Pr: A-2s(a and a’) / Mastic: D-? / Exo: 4l / E: D-3l

T2 (Fig. 7C, D) a walking limb. Protopodite with seta d2 of medium length, seta d1 absent. First two endopodal segments with one long antero-apical seta each. Seta e reaching half of the penultimate segment and seta f reaching tip of terminal segment. Third endopodal segment with two antero-apical setae, one medium length g-seta and one very short seta. Fourth segment with one very short seta (h3), one short seta (h1) and long, strongly curved and serrated claw (h2); distal claw ~ 3× as long as the second endopodal segment.

Chaetotaxic formula: Pr: A-1m(d2) / EI: A-1l / EII: A-1l / EIII: A-1m-1s / EIV: P-1s(h3), D-1s(h1)-1l(h2 G:ser)

T3 (Fig. 7E) a cleaning limb. Protopodite with two long setae (dp and d2) and one shorter seta (d1). First endopodal segment with short subapical e-seta. Second and third endopodal segments fused, with short f-seta not reaching tip of segment. Terminal part (fusion between distal part of third and fourth segment) a pincer organ, bearing a medium length seta (h3), a short serrated seta (h2), and a very short seta (h1), length of seta h3 ~ 2/3 that of fused segment, seta h2 ~ 1/2 length of seta h3.

Chaetotaxic formula: Pr: A-1l(d2)-1m(d1), P-1l(dp) / EI: A-1s(e) / EII + III: A-1s(f) / EIV: 1s(h2: ser)-1m(h3)

CR (Fig. 7F). Reduced, with elongated, triangular base; distally with long flagellum-like seta and subapically with a short seta.

**Measurements** (in μm). Cp (n = 3): L = 433–464, H = 259–282; LV (n = 6): L = 430–461, H = 250–272; RV (n = 6): L = 444–473, H = 261–275.

**Male** unknown.

##### Ecology.

*Sarscypridopsisharundineti* was found in eight temporary waterbodies of the vast floodplains south of the Okavango Delta in northern Botswana. Habitats include both lotic (river side channel, floodplain channel) and lentic waters (flooded swamp, grassland, isolated pool) as well as the endorheic Lake Ngami. The species occurred at the pH range of 6.5–7.7, the electrical conductivity range of 102–464 µS/cm, and the water temperature range of 19.8–33.7 °C.

## ﻿Discussion

### ﻿*Potamocypris* and *Cyprilla* Sars, 1924

Sars (1924a) described the genus *Cyprilla*, based on the carapace shape and presence of large flanges on the left valve, causing a noticeable LV > RV overlap. Five species were assigned to this genus, all raised from dry mud or obtained from water samples, from South Africa: *C.arcuata* Sars, 1924, *C.deflexa* Sars, 1924, *C.gibbula* Sars, 1924, *C.humilis* Sars, 1924 and *C.producta* Sars, 1924. In the same publication, Sars (1924a) noticed that *Cyprilla* differs from *Potamocypris* “in the general appearance of the shell and in the mutual relation of the valves, as also apparently in the sculpture”, but shares with *Potamocypris* reduced caudal rami and a spatulate terminal segment of the Mx1 palp. According to Gauthier (1939), these features cannot be considered diagnostic, and thus he transferred the five *Cyprilla* species to *Potamocypris*, automatically synonymising *Cyprilla* with *Potamocypris*. The same view was supported by Meisch (1984, 1985) and George and Martens (2002). While redescribing *P.humilis* from the Outer Hebrides off the west coast of Scotland, Horne and Smith (2004) noticed prominent tubercules on juvenile carapaces, the trait not yet described in any *Potamocypris*. They partly agreed with Purasjoki (1948) who considered the presence of tubercules in juveniles a *Cyprilla* trait, in this way questioning the previously proposed synonymisation of the two genera. This merits a further study, but we confirm that none of the juveniles of *P.meissneri* we collected had tubercules. Therefore, we feel confident in describing the new species as a member of *Potamocypris*.

*Potamocyprismeissneri* differs from other species of the genus by the presence of a conspicuously reticulate carapace, densely covered by prominent conuli carrying rimmed pores with long extending sensilla, and by wide anterior and posterior flanges on the left valve. Out of five southern African *Potamocypris* species only one, *P.paludum* Gauthier, 1939 (nom. nov. pro *Cyprillaarcuata* Sars, 1924 nec Sars, 1903 – *fide* Gauthier, 1939), has short swimming setae on the second antennae, which clearly distinguishes it from the new species described here. Two further species, *P.mastigophora* (of which *Cyprillaproducta* Sars, 1924 is a synonym – *fide* McKenzie, 1971) and *P.gibbula* have different carapace shapes from *P.meissneri*: more elongated and crescent-shaped in *P.mastigophora*, and more subtriangular, and dorsally arched with a blunt angle in *P.gibbula*. The remaining two species (i.e., *P.humilis* and *P.deflexa*) are more similar to *Potamocyprismeissneri* as they also have ornamented carapaces. However, unlike in *Potamocyprismeissneri*, the posterior part of the dorsal margin in the two species is almost straight, sloping down and making a distinct angle with the characteristically truncated and almost straight posterior margin. In addition, none of the presently known species of *Potamocypris* has this type of pronounced external valve ornamentation. *Potamocyprisnarayanani* George & Martens, 2002 carries conspicuous stiff setae and has a pitted valve surface, but lacks the prominent ridges and has a huge dorsal hump on the left valve.

### ﻿*Sarscypridopsis*

The originally assigned type species, *S.gregaria* (Sars, 1895), was placed into the synonymy of *S.aculeata* (Costa, 1847) by Sywula (1966), and both were originally assigned to the genus *Cypridopsis*, which originally included all cypridinid ostracods with a flagellate CR. However, over time, as more species with this trait had been found, new genera were distinguished, *Sarscypridopsis* being one of those. McKenzie (1977) ignored the synonymy proposed by Sywula (1966) and made *S.gregaria* the type species of his genus *Sarscypridopsis*. The status of *S.gregaria* has to be rechecked on type material, to clearly determine which is the true type species of this genus: *S.aculeata* with *S.gregaria* being its junior synonym, or *S.gregaria* as a valid species. Nominally, of course, *S.gregaria* will always remain the designated type species.

According to McKenzie (1977) and Meisch (2000)*Sarscypridopsis* is diagnosed by the following morphological characters: carapace rather small (< 0.9 mm), subtriangular in shape with smooth or pitted surface; RV overlapping LV ventrally, anteriorly and posteriorly; calcified inner lamella broad anteriorly and narrower posteriorly; distal segment of Mx1 palp cylindrical and ramus of the CR triangular. As our new species show all of these characteristics, we herewith assign it to the genus *Sarscypridopsis*. However, compared to other congeners, the base of the CR is unusually elongated.

*Sarscypridopsis* is mostly an Afrotropical genus (Meisch et al. 2019). Of the 17 species presently assigned to this genus, only three are known to occur also outside the African continent. *Sarscypridopsisaculeata* is nearly cosmopolitan (Meisch et al. 2019), *S.lanzarotensis* (Mallwitz, 1984) has been found in Spain including Canary Islands (Mallwitz 1984; Meisch 2000; Castillo-Escrivà et al. 2016), Italy (Pieri et al. 2015), and Morocco (Scharf and Meisch 2014), while *S.ochracea* (Sars, 1924), except for the Afrotropical region, has also been reported from the Oriental and Australian regions (Meisch et al. 2019), although these latter identifications seem unlikely from a zoogeographical point of view and need confirmation.

Most of the *Sarscypridopsis* species are poorly described and only for the following three species the morphology of the soft parts is (partly) known: *S.aculeata*, *S.katesae* (Hartmann, 1957) and *S.lanzarotensis*. In all three of these species, the respiratory plate of T1 carries five rays, while *S.harundineti* has only four rays, although admittedly this character is often very difficult to observe. Another difference between the new species and *S.aculeata* and *S.lanzarotensis* is the presence of two smooth teeth bristles on the third endite of Mx1 in *S.harundineti*. In the other two species the proximal bristle is serrated as is the neighboring one (Mallwitz 1984; Meisch 2000). In *Sarscypridopsiskatesae* these teeth bristles are also smooth (Hartmann 1957).

Despite of the lack of comparative characters, *Sarscypridopsisharundineti* can be easily distinguished from its South African congeners by the unique, more rounded valves shape, and the smaller carapace (L = 0.43–0.47 mm versus 0.54–0.80 mm). The greatest height is situated just behind mid-length in the new species, unlike in *S.clavata* (Sars, 1924), *S.echinata* (G.W. Müller, 1908), *S.elizabethae*, *S.hirsuta* (Sars, 1924), *S.punctata* (Sars, 1924), *S.reniformis* (Sars, 1924) and *S.striolata* (Sars, 1924) in which the dorsal margin of the carapace is more arched and the greatest height is situated more to the front. *Sarscypridopsisochracea* (Sars, 1924), *S.pyramidata* (Sars, 1924), *S.tonsa* (Sars, 1924) and *S.trigonella* (Sars, 1924) have sub-triangular carapaces, while *S.glabrata* (Sars, 1924) has more elongated one. The carapace size and the shape in the lateral view of *Sarscypridopsisharundineti* is most similar to *S.brevis* (Sars, 1924) and *S.sarsi* (Klie, 1935). The former can be distinguished by a very hirsute external surface of the carapace, while the latter has a distinctly globular carapace in dorsal view (Sars 1924a, 1924b).

Taking into account the mentioned gaps in information on taxonomic traits, we conclude that southern African Cypridopsinae, especially representatives of the genus *Sarscypridopsis*, urgently need integrated taxonomic revision, i.e., by means of both morphological characters (including redescriptions based on both the type and newly collected material) and DNA-sequence data.

## Supplementary Material

XML Treatment for Potamocypris meissneri

XML Treatment for Sarscypridopsis harundineti

## Supplementary materials

Supplementary material 1 Pictures of the sites from where new species *Potamocyprismeissneri* sp. nov. and *Sarscypridopsisharundineti* sp. nov. were collected Data type Images Explanation note Pictures of the sites from where *Potamocyprismeissneri* sp. nov. and *Sarscypridopsisharundineti* sp. nov. were collected in the North-West Province of South Africa (A) and in the floodplains south of the Okavango Delta near the town of Maun (except C) in the North-West District of Botswana (B-I), respectively. **A** SA-9: a small temporary open pan near the village of Ganalaagte **B** SA-103: grassy shore of a seasonal pond **C** SA-96: endorheic Lake Ngami **D** SA-97: Thamalakane river **E** SA-98: a temporary channel **F** SA-99: a temporary channel **G** SA-100: a flooded swamp and grassland **H** SA-101: an isolated pool in flooded grassland **I** SA-102: a floodplain channel. https://binary.pensoft.net/file/621026This dataset is made available under the Open Database License (http://opendatacommons.org/licenses/odbl/1.0/). The Open Database License (ODbL) is a license agreement intended to allow users to freely share, modify, and use this Dataset while maintaining this same freedom for others, provided that the original source and author(s) are credited.A. Szwarc, K. Martens, T. Namiotko
