# Supplementary figures and images for: ﻿Two new Cypridopsinae Kaufmann, 1900 (Crustacea, Ostracoda) from southern Africa
Source: Zookeys. 2021 Dec 9;1076:83–107. doi: 10.3897/zookeys.1076.76123 (PMC8677703; doi:10.3897/zookeys.1076.76123)

A

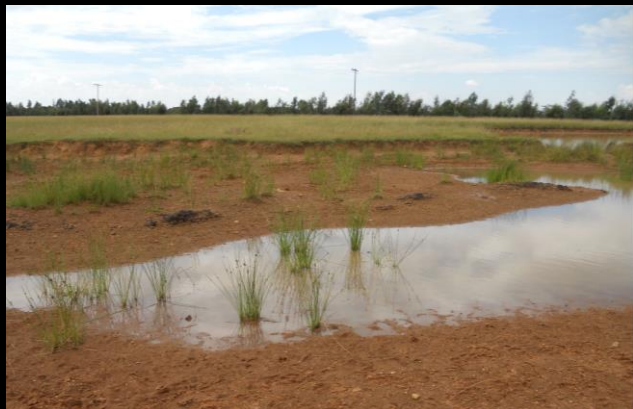

B

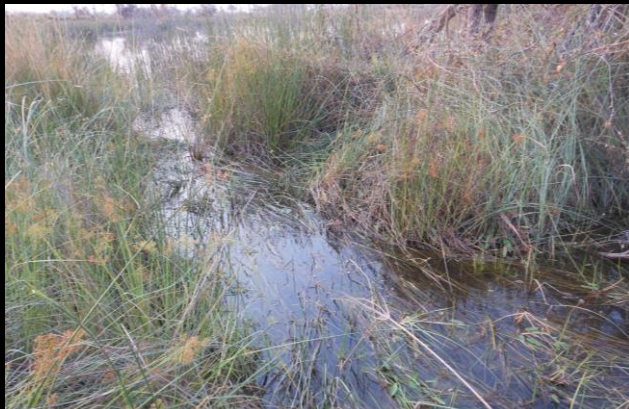

C

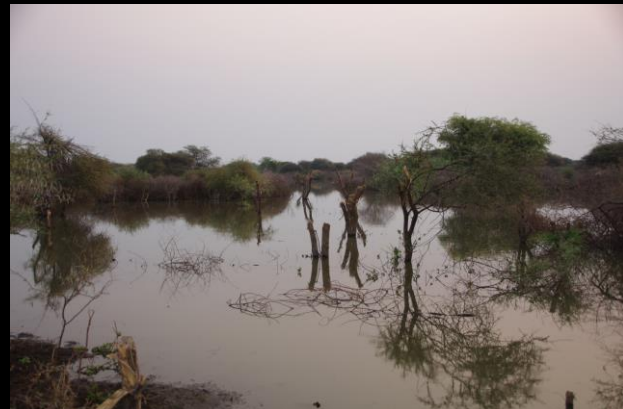

D

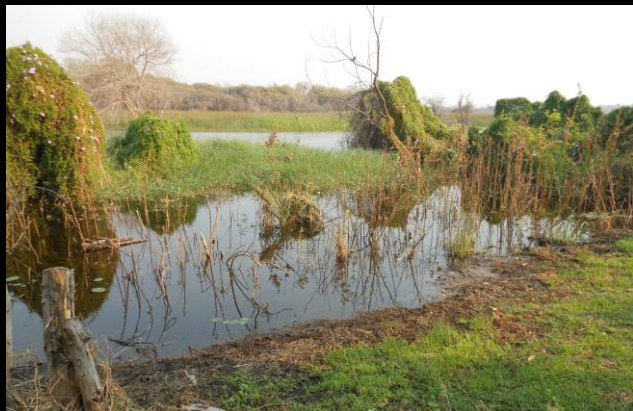

E

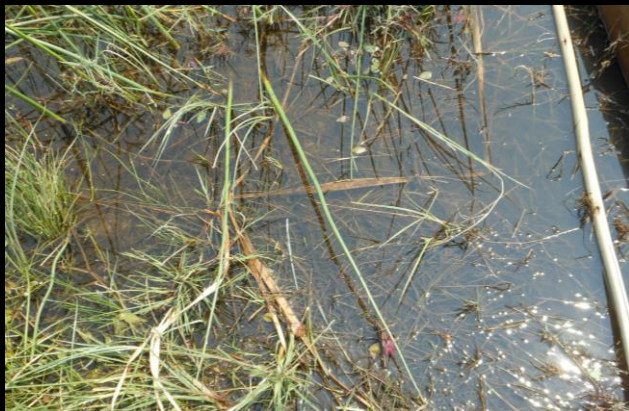

F

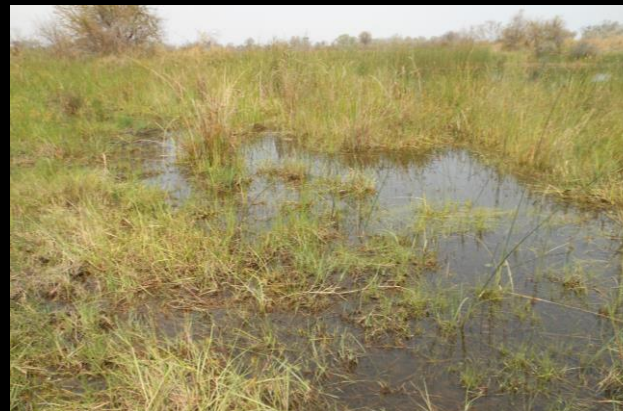

G

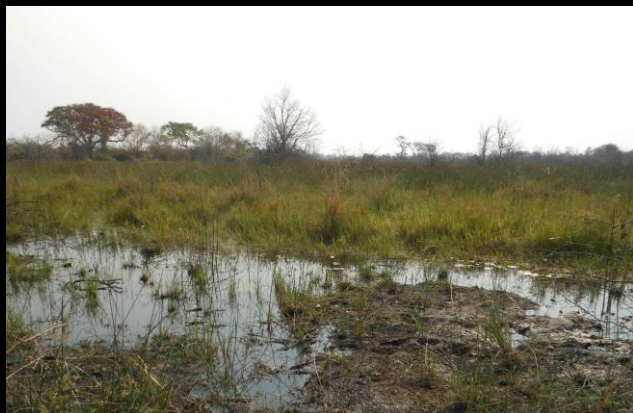

H

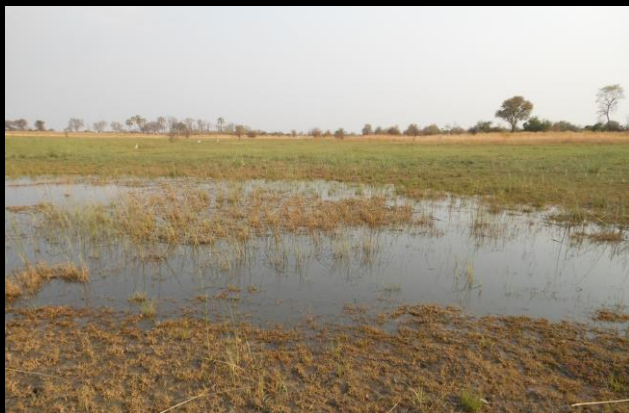

I

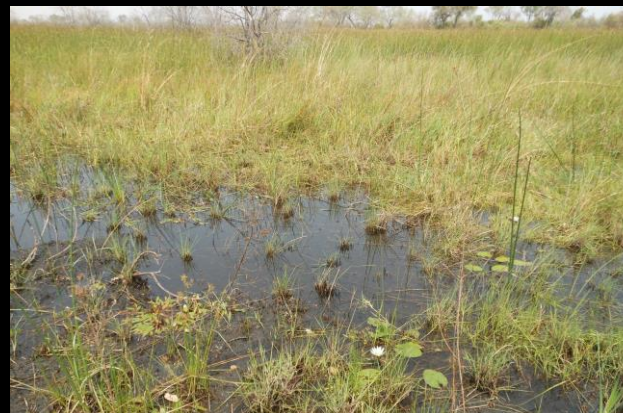

Supplement: Supplementary material 1 — Pictures of the sites from where new species Potamocyprismeissneri sp. nov. and Sarscypridopsisharundineti sp. nov. were collected [file zookeys-1076-083-s001.pdf]
